# Surveillance of laboratory exposures to human pathogens and toxins, Canada, 2023

**DOI:** 10.14745/ccdr.v51i01a03

**Published:** 2025-01-02

**Authors:** Abdulwadud Nafees, Audrey Gauthier, Antoinette N Davis, Emily F Tran, Christine Abalos, Christa M Girincuti, Samuel Bonti-Ankomah

**Affiliations:** 1 Regulatory, Operations and Emergency Management Branch, Public Health Agency of Canada, Ottawa, ON

**Keywords:** Centre for Biosecurity, human pathogens and toxins, laboratory-acquired infections, laboratory exposures, laboratory incidents, Laboratory Incident Notification Canada, surveillance

## Abstract

**Background:**

The Public Health Agency of Canada oversees the *Human Pathogens and Toxins Act* and *Human Pathogens and Toxins Regulations*, and monitors human pathogen and toxin incidents in licensed facilities to minimize exposure impact at the individual and population level.

**Objective:**

To provide an overview of confirmed laboratory exposure incidents in Canada in 2023.

**Methods:**

Confirmed exposure incident reports in 2023 were analyzed using R 4.2.2, Microsoft Excel and SAS.

**Results:**

In 2023, 207 incident reports were received, including 63 confirmed exposure incidents that affected 85 individuals. The academic sector accounted for 50.8% (n=32) of the reported confirmed exposure incidents. Microbiology (n=33; 52.4%) was the predominant activity being performed, with the most common occurrence types being sharps-related (n=22; 27.2%) and procedure-related (n=16; 19.8%). Human interaction (n=36; 57.1%) and standard operating procedures (n=24; 38.1%) were the most frequent root causes cited, with corrective actions often directly addressing these causes. Most of the 85 affected individuals were technicians/technologists (n=55; 64.7%) and had a median of 11 years of laboratory experience. Sixty-seven human pathogens and toxins (HPTs) were implicated in the confirmed exposure incidents, with bacteria (n=36; 53.7%) being the most common biological agent type. The median time between the incident and the reporting date was six days.

**Conclusion:**

The number of confirmed exposure incidents increased in 2023 compared to 2022. Microbiology was most often the activity being performed at the time of exposure, and occurrence-types, root causes and HPTs implicated in 2023 mirrored those cited in 2022.

## Introduction

In the field of biosafety, the management of human pathogens and toxins (HPTs) is a matter of importance due to the potential for laboratory-acquired infections (LAIs) ((1–6)). Recognizing this risk, a rigorous approach to biosafety in facilities where controlled activities are conducted is necessary, including regulated safety practices and incident surveillance.

The backbone of Canada's regulatory framework in laboratory safety is the *Human Pathogens and Toxins Act* (HPTA) ((7)) and the *Human Pathogens and Toxins Regulations* (HPTR) ((8)), which are administered and enforced by the Public Health Agency of Canada's (PHAC's) Centre for Biosecurity. Since the enactment of the HPTA in 2009 and the HPTR in 2015, the HPTA/HPTR have set the standards for working with HPTs in various sectors such as hospitals, academic institutions and public or private institutions in Canada. There are four risk groups that classify HPTs based on their potential to harm individual and community health. For instance, risk group 1 (RG1) HPTs, like non-pathogenic *Escherichia coli*, are not expected to cause disease in humans, while risk group 4 (RG4) HPTs, such as the Ebola virus, are known for their potential to cause life-threatening diseases that spread rapidly through the community ((9)). Also included amongst these regulated HPTs are a class of risk group 3 (RG3) and RG4 HPTs known as security sensitive biological agents (SSBAs) that are specified due to their potential for use as biological weapons and for bioterrorism ((9)).

The Centre for Biosecurity established the Laboratory Incident Notification Canada (LINC) surveillance system in late 2015 to oversee HPT incident reporting, identification, monitoring and analysis and ensure appropriate follow-up and support to licensed facilities with the goal of reducing the risk of recurrence ((10)) and minimizing the impact of exposures on the health and wellbeing of facility personnel and the general population. Compared to incident surveillance systems in other developed countries, LINC remains the most comprehensive in terms of its scope. For instance, both the Federal Select Agent Program ((11)) in the United States and the Security Sensitive Biological Agents Standards ((12)) in Australia were established to provide regulatory oversight for only SSBAs, with the former producing an annual report on its inspections, compliance actions, transfer of biological select agents and toxins as well as the theft, loss or release of biological select agents and toxins in order to improve understanding of their mandate ((13)). Operating under the HPTA and HPTR, LINC's scope includes a much broader range of HPTs and is not limited to SSBAs ((14)).

Under the HPTA, any facility working with risk group 2 (RG2) pathogens and above must obtain a pathogen and toxin licence to conduct controlled activities with HPTs ((7,15)). The licence requires facilities to adhere to the outlined safety protocols and reporting standards. Licensed facilities are mandated to report various types of incidents to LINC without delay, including exposure incidents, which involve potential or actual contact with pathogens, and non-exposure incidents, such as a missing, lost or stolen biological agent, the inadvertent possession, production or release of an HPT and SSBAs not received at the facility within their expected arrival time. Other incidents that must be reported without delay include changes to biocontainment and other biosafety-related occurrences that may not directly involve pathogen exposure but have significant implications for laboratory safety. The reporting of incidents involving agents in their natural environment is voluntary. Pathogens in their natural environment refer to those present in uncultured or unprocessed samples collected directly from humans or animals. Such biological materials may include blood, serum, saliva, milk or urine.

The year 2023 marked the eighth year of the LINC surveillance system. The program's duration has allowed for the meaningful analysis of incident data from more than 361 confirmed exposure reports ((16)), which provided insight into laboratory safety measures, highlighted areas of progress and ongoing challenges ((10,14,17–21)) and illuminated exposure incident trends such as the most common biological agent types (bacteria and virus) and leading root causes (standard operating procedures [SOPs] and human interaction).

This report summarizes exposure incidents in Canada that were reported to LINC in 2023 with the goal of enhancing awareness of the risks associated with handling HPTs, informing biosafety measures in facilities and comparing the incident data to those from previous years.

## Methods

### Data sources

Laboratory Incident Notification Canada is the Government of Canada's primary mechanism for collecting and monitoring incidents involving HPTs in licensed facilities across Canada under the HPTA and the HPTR. This system, which is accessible through an online Biosecurity Portal, facilitates the reporting of exposure, non-exposure and other types of incidents by licensed facilities. Once reported, these incidents are viewed and processed by LINC in the Integrated Suite of Tools for Operational Processes (iSTOP) of the Microsoft Customer Relationship Management system.

When a licensed facility reports an exposure incident, they are required to submit one or more follow-up reports in addition to their initial exposure report in order to provide further details and the most updated information regarding the incident.

Incidents reported between January 1, 2023, and December 31, 2023, were extracted from iSTOP on February 6, 2024, and analyzed. The analysis included incidents without a specified occurrence date, provided they were reported within this timeframe. Utilizing only the most recent follow-up reports ensured that the analysis was based on the latest and most accurate information pertaining to each incident. In cases where follow-up reports were not yet submitted to LINC, initial exposure report data were used. The extraction process involved examination for outliers and the removal of any duplicate entries to maintain the integrity of the data. The total number of active licences was extracted from the Customer Relationship Management on February 18, 2024, and additional filters were applied in iSTOP to obtain the number of active licences per sector. Some licences did not have a specified sector.

### Report variables

The following variables were used to describe the confirmed exposure reports: the main activity being performed at the time of the exposure incident; sector affected; individuals, pathogens and toxins involved; root causes and corrective actions; occurrence types; and time delay in reporting. The definitions for the main activities are provided in **Appendix Table A1**. Sector variables include nine categories: academic; hospital; public health; veterinary/animal health; private industry/business; other government; environmental health; not specified; and “do-it-yourself biology,” where “do-it-yourself biology” refers to any individual not working in an institutionalized facility who is conducting their own experiments. Information about affected individuals, such as their role, years of experience and highest level of education, was also collected. Data on other characteristics, such as their age, gender and socioeconomic status, were not collected.

### Data analysis

This report focuses on the confirmed exposure incidents reported to LINC in 2023. The classification of incidents into confirmed or ruled-out categories was based on a review of follow-up reports. Data were run in R 4.2.2 software for data wrangling, cleaning and generating descriptive statistics. Microsoft Excel and SAS 9.4 were used for data validation and to generate figures and tables. This dual approach allowed for cross-validation and ensured the quality of data for analysis. This year's analysis also re-examined data from 2016–2022 to account for any updates to previously submitted reports.

The exposure incident rate per 1,000 active licences was calculated by comparing the total number of reported exposure incidents against the total number of active licences during the surveillance period, multiplied by 1,000, to provide a standardized measure to assess trends over time and across different regulatory sectors.

### Baseline establishment

An annual and monthly average of exposure incidents from 2016–2022 was calculated along with 95% confidence intervals using Microsoft Excel. To establish an annual baseline incidence, data from 2016–2022 were pooled and the total number of confirmed exposure incidents from 2016–2022 was summed and divided by the total number of active licences from 2016–2022 and multiplied by 1,000 to obtain the annual baseline incidence of exposures per 1,000 active licences.

## Results

**Figure 1:**
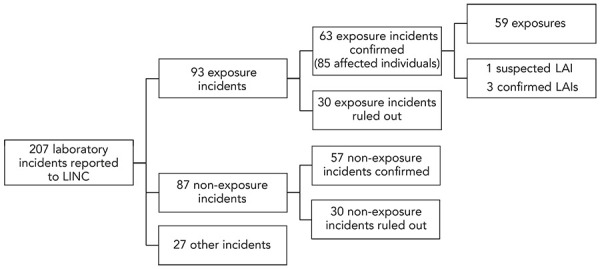
Incidents reported to Laboratory Incident Notification Canada, 2023 Abbreviations: LAI, laboratory-acquired infection; LINC, Laboratory Incidence Notification Canada

**Figure 1** depicts the 207 laboratory incident reports submitted to LINC from January 1, 2023, to December 31, 2023. Out of these, 93 (44.9%) were exposure reports, 87 (42.0%) were non-exposure reports and 27 (13.0%) were other reports. Thirty exposure reports and 30 non-exposure reports were ruled out, leaving 63 confirmed exposure incidents with 85 affected individuals in 2023. Amongst the confirmed exposure incidents, there was one suspected LAI and three confirmed LAIs.

**Figure 2:**
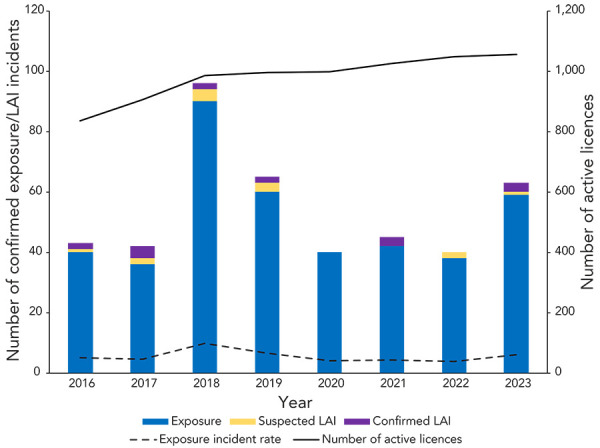
Confirmed exposure incidents, suspected and confirmed laboratory-acquired infections, active licences and exposure incident rate, 2016–2023 Abbreviation: LAI, laboratory-acquired infection

There was a total of 1,057 active licences (**Figure 2**) in 2023, including 981 licences for RG2 HPTs, 70 licences for RG3 pathogens, two licences for RG4 pathogens and four licences for SSBAs. The number of confirmed exposure incidents per 1,000 active licences (the exposure incident rate) was 60. From 2016–2022, there was an average of 53.0 (95% CI: 38.7–7.3) exposure incidents per year and a yearly baseline incidence of 54.6 exposure incidents per 1,000 active licences.

**Figure 3:**
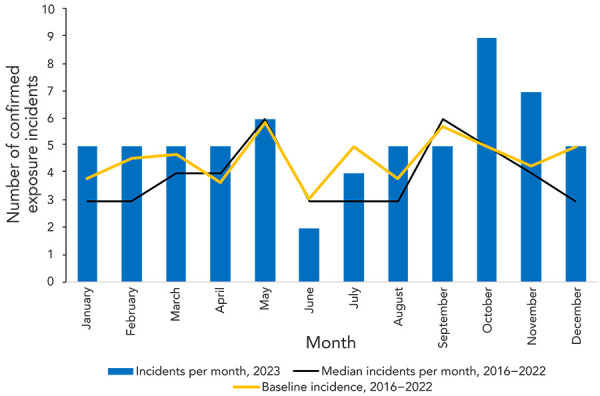
Seasonality analysis using median confirmed exposure incidents per month and baseline incidence, 2016–2023

From 2016–2022, there was an average of 4.4 (95% CI: 3.8–5.0) exposure incidents per month. The number of confirmed exposure incidents remained relatively stable in 2023, with five confirmed exposure reports each month for seven of the 12 months (**Figure 3**). The lowest number of exposure reports occurred in June (n=2; 3.2%) and the highest occurred in October (n=9; 14.3%). In comparison, the baseline incidence per month per 1,000 active licences and the median from 2016–2022 peaked in May and September.

### Exposure incidents by main activity and sector

Microbiology and *in vivo* animal research were the most common main activities being performed at the time of the confirmed exposure incident (n=33; 52.4% and n=13; 20.6%, respectively) (data not shown). Other activities (n=6; 9.5%), cell culture (n=5; 7.9%), maintenance (n=3; 4.8%), microscopy (n=2; 3.2%) and education or training (n=1; 1.6%) were also mentioned as main activities being performed at the time of exposure.

**Figure 4:**
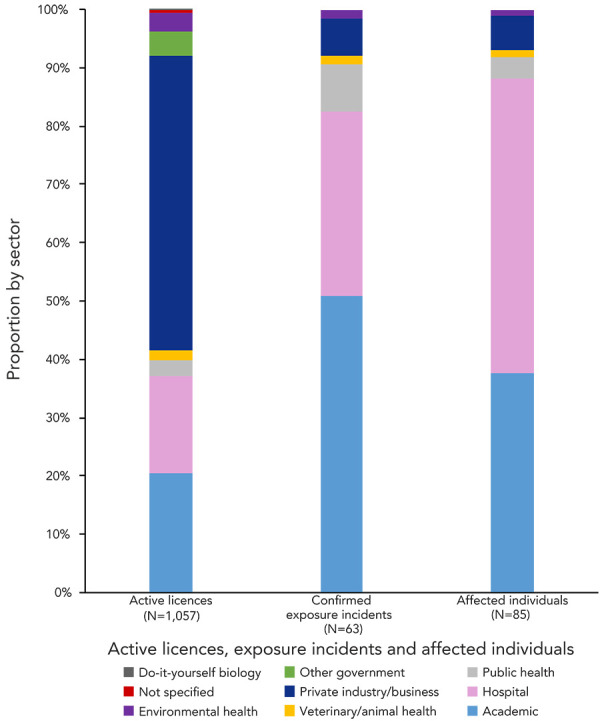
Active licences, confirmed exposure incidents and affected individuals by sector, 2023

The largest number of confirmed exposure incidents were reported by the academic (n=32; 50.8%) and hospital (n=20; 31.7%) sectors, as shown on **Figure 4**. Only four confirmed exposures were reported from the private sector (6.3%). The active licences are distributed among multiple sectors, including academic, hospital, private and public health. Most licences in 2023 were held by private facilities (n=533; 50.7%), academic facilities (n=216; 20.5%) and hospitals (n=177; 16.8%).

### Affected individuals

An average of 1.57 persons were affected per confirmed exposure incident in 2023, with 85 individuals affected in total. Of these 85 individuals, 43 were affected through confirmed exposure incidents in hospital sector (50.6%), while 32 were affected through confirmed exposure incidents in the academic sector (37.6%), as shown in Figure 4. The veterinary/animal health and environmental health sectors each had one confirmed exposure (1.6%) with one affected individual (1.2%) in each.

**Figure 5:**
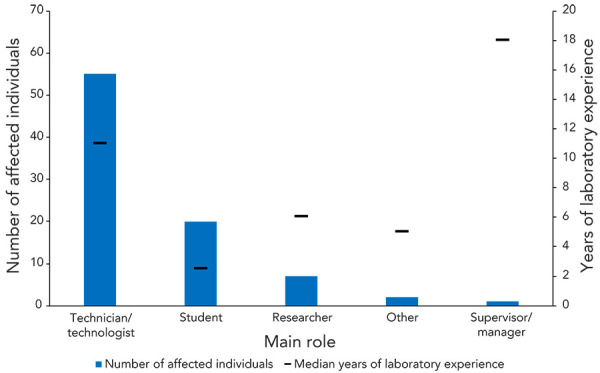
Affected individuals in confirmed exposure incidents reported by number of years of laboratory experience and main role, 2023 (N=85)

The largest number of individuals affected in a single confirmed exposure incident (inhalation of *Brucella melitensis* caused by an inadvertent possession of the pathogen) was 11 in a hospital laboratory. The majority of individuals affected in confirmed exposure incidents in 2023 were technicians/technologists (n=55; 64.7%) with a median number of 11 years of experience working in a laboratory setting (**Figure 5**). Among the affected individuals, 20 were students (23.5%) with a median of 2.5 years of experience and seven were researchers (8.2%) with a median of six years of experience. In 2023, only one supervisor/manager was involved in a confirmed exposure incident (1.2%). That individual had 18 years of laboratory experience.

### Implicated human pathogens and toxins

Sixty-seven HPTs were implicated in confirmed exposure incidents in 2023 (**Table 1**). Exposures were predominantly with non-SSBAs (n=57; 85.1%). Among the RG2 HPTs (n=48; 71.6%), the most common agent types were bacteria (n=30; 44.8%) and viruses (n=14; 20.9%). Other HPT agent types, such as fungus, parasite, prion and cell line, were each implicated in one exposure incident. For exposure incidents involving RG3 HPTs (n=15; 22.4%), the most common agent types were bacteria (n=6; 9.0%), fungus (n=5; 7.5%) and virus (n=3; 4.5%). The RG2 HPTs most frequently implicated in exposure incidents were *Neisseria meningitidis* (n=8; 16.7%) and *Staphylococcus aureus* (n=7; 14.6%), while among the RG3 agents, *B. melitensis* (n=3; 20%) as well as *Histoplasma capsulatum* and *Mycobacterium tuberculosis* (n=2; 13.3% each) were most common. Only one exposure incident implicating SARS-CoV-2 was reported in 2023. Enterohemorrhagic *E. coli* and *Salmonella enterica* were implicated in two of the three confirmed LAIs, while the HPT implicated in the third confirmed LAI was unknown. Shiga toxin-producing *E. coli* (STEC) was implicated in the suspected LAI. There were no exposures to RG4 pathogens in 2023.

**Table 1:** Human pathogens and toxins implicated in reported exposure incidents by risk group level and biological agent security sensitive status, 2023 (N=67)

Biological agent type by risk group	Non-SSBA		SSBA		Unknown		Total	
	n	%	n	%	n	%	n	%
**RG2**	**48**	**71.6**	**0**	**0**	**0**	**0**	**48**	**71.6**
Bacteria	30	44.8	0	0	0	0	30	44.8
Fungus	1	1.5	0	0	0	0	1	1.5
Parasite	1	1.5	0	0	0	0	1	1.5
Prion	1	1.5	0	0	0	0	1	1.5
Toxin	0	0	0	0	0	0	0	0
Virus	14	20.9	0	0	0	0	14	20.9
Cell line	1	1.5	0	0	0	0	1	1.5
**RG3**	**9**	**13.4**	**6**	**9.0**	**0**	**0**	**15**	**22.4**
Bacteria	2	3.0	4	6.0	0	0	6	9.0
Fungus	4	6.0	1	1.5	0	0	5	7.5
Parasite	0	0	0	0	0	0	0	0
Prion	1	1.5	0	0	0	0	1	1.5
Toxin	0	0	0	0	0	0	0	0
Virus	2	3.0	1	1.5	0	0	3	4.5
Cell line	0	0	0	0	0	0	0	0
**Unknown agents**	**0**	**0**	**0**	**0**	**4**	**6.0**	**4**	**6.0**
**Total**	**57**	**85.1**	**6**	**9.0**	**4**	**6.0**	**67**	**100**

Abbreviations: RG2, risk group 2; RG3, risk group 3; SSBA, security sensitive biological agents

### Occurrence types

**Figure 6:**
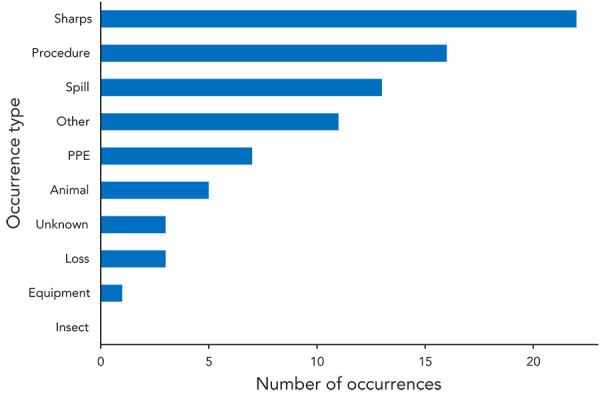
Occurrence types involved in confirmed exposure incidents, 2023 (N=81) Abbreviations: animal, animal-related; equipment, equipment-related; loss, loss of containment-related; PPE, personal protective equipment-related; procedure, procedure-related; sharps, sharps-related

More than one occurrence type could be selected for each of the 63 confirmed exposure incidents. Eighty-one occurrence types were identified in 2023 (**Figure 6**). The most frequently cited occurrence type was sharps-related (n=22; 27.2%). There were also 16 (19.8%) procedure-related occurrences, 13 (16.0%) spill-related occurrences and 11 (13.6%) occurrences categorized as “other.” The “other” occurrence type included exposures due to work performed on an open bench and accidental ingestion. There were three (3.7%) unknown occurrence types. Definitions of the occurrence types are provided in Appendix **Table A2**.

### Root causes and corrective actions

Many of the confirmed exposure incidents were associated with more than one root cause (**Table 2**), with a total of 131 root causes identified and an average of 2.08 per exposure incident. Human interaction was the root cause identified in 36 (57.1%) confirmed exposure incidents, while SOPs were identified as the root cause in 24 (38.1%) confirmed exposure incidents.

**Table 2:** Root causes and corrective actions reported in follow-up reports of confirmed exposure incidents, 2023 (N=131)

Root cause	Examples of areas of concern	Citations		Corrective actions	
		n	%^a^	n^b^	%^c^
Human interaction	A violation (cutting a corner, not follow correct procedure, deviating from standard operating procedure)	36	57.1	22	61.1
	An error (a mistake, lapse of concentration or slip of any kind)				
Standard operating procedure (SOP)	Documents were followed as written but not correct for activity/task	24	38.1	20	83.3
	Procedures that should have been in place were not in place				
	Documents were not followed correctly				
Training	Training was not in place but should have been in place	19	30.2	15	78.9
	Training was not appropriate for task/activity				
	Staff were not qualified or proficient in performing task				
Management and oversight	Supervision needed improvement	17	27.0	11	64.7
	Lack of auditing of standards, policies and procedures				
	Risk assessment needed improvement				
Equipment	Equipment quality control needed improvement	16	25.4	8	50.0
	Equipment failed				
	Equipment was not appropriate for purpose				
Communication	Communication did not occur but should have	15	23.8	12	80.0
	Communication was unclear, ambiguous, etc.				
Other	Not applicable	4	6.3	0	0

^a^ Percentage of exposure incidents that were associated with this root cause

^b^ Number of exposures that were associated with this root cause, with the corrective action addressing the same area of concern

^c^ Percentage of exposures that were associated with this root cause, with the corrective action addressing the same area of concern

Corrective actions were compared with the root causes of each confirmed exposure incident (Table 2). The corrective actions that addressed the same root cause were related to SOPs (n=20; 83.3%), communication (n=12; 80.0%) and training (n=15; 78.9%). Only 50.0% of confirmed exposure incidents with an equipment-related root cause were addressed by corrective actions in this same area of concern (n=8).

### Reporting delay to Public Health Agency of Canada

**Figure 7:**
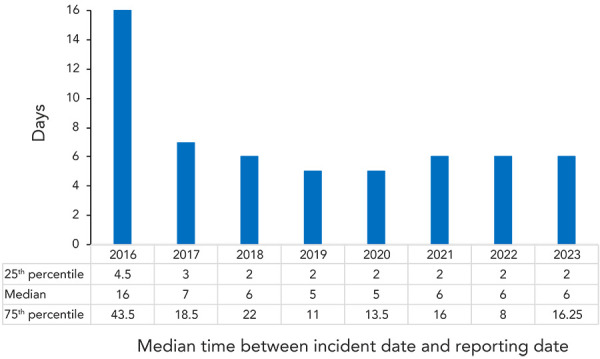
Time between the date of the confirmed exposure incident and the date it was reported to Laboratory Incident Notification Canada, 2016–2023

The reporting delay refers to the number of days between the date of the confirmed exposure incident's occurrence and the date on which it was first reported to PHAC via LINC. In 2023, the median reporting delay was six days, as was the median reporting delay in 2021 and 2022 (**Figure 7**). The 25^th^ percentile for reporting delay was two days, consistent with the previous five years, while the 75^th^ percentile was 16.25 days, more than double what it was in 2022 due to retrospective data entry of previously unreported exposure incident reports from 2016–2023 that were discovered during an on-site inspection.

## Discussion

In 2023, an increase in confirmed exposure incidents was observed in comparison with the preceding three years. While there are likely multiple contributing factors to this increase, one of the most significant may be the COVID-19 pandemic. The pandemic, which occurred between 2020 and 2022, significantly altered normal work practices in many fields by limiting the number of workers and changing the volume and type of laboratory activity conducted ((22)). The number of confirmed exposure reports in 2023 was similar to the pre-pandemic period. As seen in previous years ((10,14,17–21)) the academic and hospital sectors contributed the largest proportion of confirmed exposure incidents. The most common activities being performed during a confirmed exposure event were microbiology and *in vivo* animal research, and confirmed exposures due to sharps-, procedure- and spill-related occurrences were most often cited, with human interaction and SOPs as the most common root causes. Technicians/technologists made up the majority of affected individuals, while non-SSBAs were implicated most frequently in confirmed exposure incidents. Compared to 2021 and 2022, there was no change in the median reporting delay.

### Corrective actions undertaken following a confirmed exposure incident

Understanding the underlying causes of incidents and developing strategies to prevent recurrence, especially for system-level failures rather than individual errors, is important ((23,24)). Part of the exposure incident follow-up process includes the reporting of corrective actions taken by regulated facilities. Corrective actions fall into the same categories as root causes, allowing for an assessment of incidents based on the appropriateness of the applied corrective actions. In 2023, the root cause that was most frequently addressed through corrective actions following a confirmed exposure incident was SOP. Well-designed systems have just as much of an impact on safety as do individual-level capabilities and errors ((23)). This understanding drives SOP changes, which generally involve modifications to workflow or communication protocols. The higher rate of corrective action may reflect the tangible nature of these procedural improvements, which can be directly implemented and monitored ((23)). Training-related solutions were also frequently observed in 2023, aligning with literature that emphasizes the important role of continuous education in mitigating errors and enhancing safety ((24)). Training addresses immediate knowledge gaps and enhances skillsets ((23)). Efforts to improve communication through corrective actions, with 80.0% of related incidents addressed in 2023, emphasize the importance of effective communication channels in laboratory settings, which are foundational for error prevention and risk mitigation once an incident has already occurred ((25)).

Corrective actions were not reported for some root causes, like “other,” which included unpredictable animal behaviour. This may indicate areas where solutions are more challenging to identify or implement. Corrective actions addressing equipment or ”other” issues may require more resource-intensive solutions or reflect a lower perceived risk ((25)).

Non-security sensitive biological agents, risk group 2 and bacteria remain the most reported human pathogen and toxin types

Since the establishment of the LINC program and incident reporting, a large proportion of pathogens implicated in confirmed exposure incidents have consistently been RG2 non-SSBAs and, most commonly, bacterial agents ((10,14,17–21)). This trend continued in 2023, with non-SSBAs implicated in 85.1% of confirmed exposure incidents and RG2 HPTs accounting for 71.6% of HPTs identified. Almost 45% of agent types involved in exposure incidents were bacteria, which reflects the findings by Blacksell *et al.* (2024), where the predominant cause of exposure incidents that resulted in LAIs was a bacterial pathogen ((1)). The consistently high percentage of RG2 HPTs involved in confirmed exposure incidents reported to LINC is likely because the majority of active licences (92.8% in 2023) are held by facilities carrying out controlled activities with RG2 HPTs. Similarly, in 2023, the majority of facilities were licensed to work with non-SSBAs, with only 0.4% of active licences granted for SSBAs, thus explaining the higher proportion of non-SSBAs implicated in confirmed exposure incidents compared to SSBAs.

### Sharps and procedure-related occurrences and support for licence holders

The leading occurrence-types cited in confirmed exposure incidents in 2023 were sharps (27.2%) and procedures (19.8%). This is consistent with annual report data from previous years ((10,14,17–21)). These occurrence types, sharps in particular, frequently occur in laboratories and have often resulted in exposure incidents ((3,10)). For example, a study using data of clinical laboratory workers from private and government health sectors in Al-Madinah, Saudi Arabia also found that sharps-related injuries were commonly experienced among the workers and were associated with a lack of biosafety training ((26)). As such, preventing needlestick and sharps-related injuries within laboratories remains crucial due to their potential to transmit pathogens ((27)).

To raise awareness of common causes of exposure incidents, mitigate the recurrence and encourage a culture of laboratory biosafety, LINC developed several new resources to support licence holders. These resources, which can be found online in the PHAC Training Portal, facilitate the dissemination of biosafety best practices and clarify reporting procedures using a variety of easily accessible formats, including videos, an e-learning course, webinars, downloadable and fillable forms and a podcast.

### Strengths and limitations

A strength of this report is that it involved a comprehensive dataset, encompassing over eight years of data. The standardized reporting forms used as part of the incident reporting process ensured uniform data collection and ensured data reliability for trend analysis and identification of biosafety challenges.

This report has several limitations. Currently, individual-level data of all laboratory workers, such as their age, sex, experience and education background, income and other sociodemographic measures, are not collected. Such data could permit detailed analyses involving inferential statistics and hypothesis-based studies focused on potential variables associated with laboratory exposure incidents. Other limitations include the small sample size and the possibility of underreporting of laboratory exposure incidents, the extent of which remains unknown. It should also be noted that licensed facilities self-identify their sector when creating a user profile in the Biosecurity Portal as part of the licensing process, and they can only select one sector, though overlap with another sector may exist in actuality. For instance, a hospital may select the academic sector as their sector because they are affiliated with a university. This should be kept in mind when interpreting the results. Finally, a lack of comparable national incident reporting surveillance systems outside of Canada made it challenging to compare the findings and trends of this report with those of other countries.

## Conclusion

In 2023, the number of confirmed exposure incidents rose and resembled levels seen prior to the COVID-19 pandemic. The most common occurrence-types, main activity being performed, root causes and HPTs implicated in confirmed exposure incidents in 2023 mirrored those cited in 2022. The natural baseline that was calculated will serve as an additional reference point for assessment in future years. Findings from this report can be used to inform biosafety practices and procedures in facilities to reduce the incidence of exposure to HPTs.

## Appendices

**Table A1:** Definitions of main activity

Main activity	Definition
Animal care	Activities such as attending to the daily care of animals and providing animals with treatment
Autopsy or necropsy	Post-mortem surgical examinations for purposes such as determining cause of death or to evaluate disease or injury for research or educational purposes
Cell culture	The process of growing cells under controlled conditions. It can also involve the removal of cells from an animal or plant
Education or training	Education or training of students and/or personnel on laboratory techniques and procedures
*In vivo* animal research	Experimentation with live, non-human animals
Maintenance	The upkeep, repair and/or routine and general cleaning of equipment and facilities
Microbiology	Activities involving the manipulation, isolation or analysis of microorganisms in their viable or infectious state
Molecular investigations	Activities involving the manipulation of genetic material from microorganisms or other infectious material for further analysis
Serology	Diagnostic examination and/or scientific study of immunological reactions and properties of blood serum
Hematology	Scientific study of the physiology of blood

**Table A2:** Definitions of occurrence types

Occurrence type	Definition
Spill	Any unintended release of an agent from its container
Loss of containment	Includes malfunction or misuse of containment devices or equipment and other type of failures that results in the agent being spilled outside of, or released from, containment
Sharps-related	Includes needle stick, cut with scalpel, blade or other sharps injury (i.e., broken glass)
Animal-related	Includes animal bites or scratches, as well as other exposure incidents resulting from animal behavior (i.e., animal movement resulting in a needle stick)
Insect-related	Includes insect bites
PPE-related	Includes either inadequate PPE for the activity or failure of the PPE in some way
Equipment-related	Includes failure of equipment, incorrect equipment for the activity or misuse of equipment
Procedure-related	Includes instances when written procedures were not followed, were incorrect for the activity or were inadequate or absent

Abbreviation: PPE, personal protective equipment
